# Synthesis of practical red fluorescent probe for cytoplasmic calcium ions with greatly improved cell-membrane permeability

**DOI:** 10.1016/j.dib.2017.04.011

**Published:** 2017-04-13

**Authors:** Kazuhisa Hirabayashi, Kenjiro Hanaoka, Takahiro Egawa, Chiaki Kobayashi, Shodai Takahashi, Toru Komatsu, Tasuku Ueno, Takuya Terai, Yuji Ikegaya, Tetsuo Nagano, Yasuteru Urano

**Affiliations:** a Graduate School of Pharmaceutical Sciences, The University of Tokyo, 7-3-1 Hongo, Bunkyo-ku, Tokyo 113-0033, Japan; b Precursory Research for Embryonic Science and Technology (PRESTO), Japan Science and Technology Agency (JST), 4-1-8 Honcho, Kawaguchi, Saitama 332-0012, Japan; c Drug Discovery Initiative, The University of Tokyo, Tokyo 113-0033, Japan; d Graduate School of Medicine, The University of Tokyo, 7-3-1, Hongo, Bunkyo-ku, Tokyo 113-0033, Japan; e CREST, AMED, Saitama 332-0012, Japan

## Abstract

In this data article, we described the detailed synthetic procedure and the experimental data for the synthesis of a red-fluorescent probe for calcium ions (Ca^2+^) with improved water solubility. This Ca^2+^ red-fluorescent probe CaTM-3 AM could be applied to fluorescence imaging of physiological Ca^2+^ concentration changes in not only live cells, but also brain slices, with high cell-membrane permeability leading to bright fluorescence in biosamples. The data provided herein are in association with the research article “The Development of Practical Red Fluorescent Probe for Cytoplasmic Calcium Ions with Greatly Improved Cell-membrane Permeability” in Cell Calcium (Hirabayashi et al., 2016) [1].

**Specifications table**

**Table:** 

Subject area	*Chemistry*
More specific subject area	*Synthesis of fluorescent probes*
Type of data	*Synthetic schemes, experimental synthesis protocols, NMR and MS spectra, HPLC chromatogram*
How data was acquired	*NMR: JNM-LA300 (JEOL) or JNM-LA400 (JEOL), mass spectroscopy: JMS-T100LC AccuTOF (JEOL), HPLC analyses: Inertsil ODS-3 (4.6 ×250 mm) column (GL Sciences Inc.) using a HPLC system composed of a pump (PU-2080, JASCO) and a detector (MD-2018 or FP-2025, JASCO)*
Data format	*Analyzed*
Experimental factors	*Starting compounds were either purchased or synthesized using already published synthetic protocols*
Experimental features	*Compounds were synthesized and their structures were identified by NMR and mass spectrometry*
Data source location	*Tokyo, Japan*
Data accessibility	*Data are provided with this article*

**Value of the data**

• The data allows to reproduce the experiments described in the research article Ref. [1].
• The synthesized compound, CaTM-3 AM, could be used to fluorescence imaging of the physiological Ca^2+^ concentration change.
• The data provides an opportunity to synthesize red fluorescent probes based on TokyoMagenta (TM) derivatives.

## 1 Data

NMR spectra were recorded on a JEOL JNM-LA300 instrument at 300 MHz for ^1^H NMR and at 75 MHz for ^13^C NMR or JEOL JNM-LA400 instrument at 100 MHz for ^13^C NMR. Mass spectra (ESI^+^) were measured with a JEOL JMS-T100LC AccuTOF for ESI. HPLC analyses were performed on an Inertsil ODS-3 (4.6×250 mm) column (GL Sciences Inc.) using a HPLC system composed of a pump (PU-2080, JASCO) and a detector (MD-2018 or FP-2025, JASCO).

## 2 Experimental design, materials and methods

### 2.1 Synthetic materials and instrumentation

General chemicals were of the best grade available, supplied by Tokyo Chemical Industries, Wako Pure Chemical, Aldrich Chemical Co., Kanto Chemical Co., Inc., Toronto Research Chemicals Inc., Watanabe Chemical Industries, Applied Biosystems, Dojindo and Invitrogen Corp., and were used without further purification. All solvents were used after appropriate distillation or purification. Preparative HPLC were performed on an Inertsil ODS-3 (10×250 mm) column (GL Sciences Inc.) using a HPLC system composed of a pump (PU-2080, JASCO) and a detector (MD-2015 or FP-2025, JASCO) or a HPLC system composed of a pump (PU-2086, JASCO) and a detector (MD-2018, JASCO).

### 2.2 Synthesis and characterization of compounds

#### 2.2.1 Synthesis of compound **1**

**Figure:**
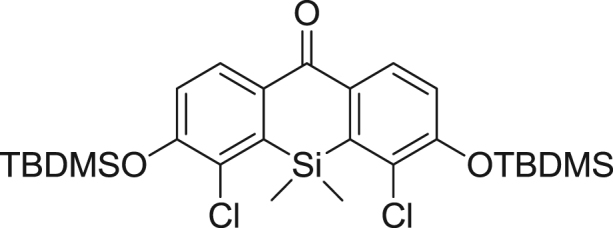

This compound was synthesized according to Ref. [2].

#### 2.2.2 Synthesis of di-tert-butyl 4-bromoisophthalate (**2**)

**Figure:**
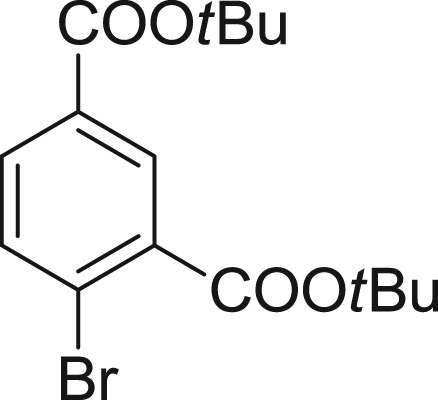

To a mixture of 4-bromoisophthalic acid (4.90 g, 20.0 mmol), *N,N*-dicyclohexylcarbodiimide (10.0 g, 46.5 mmol) and *N,N*-dimethylaminopyridine (500 mg, 4.10 mmol) in CH_2_Cl_2_ (100 mL), *tert*-butyl alcohol (50 mL) was added. The mixture was stirred at room temperature overnight. The precipitate was filtered off, and the filtrate was evaporated to dryness. The residue was purified by column chromatography (silica gel, 1/1 CH_2_Cl_2_/hexane) to give di-*tert*-butyl 4-bromoisophthalate (**2**) (4.32 g, 61% yield). ^1^H NMR (300 MHz, CDCl_3_): δ 1.60 (s, 9H), 1.62 (s, 9H), 7.67 (d, 1H, *J* = 8.1 Hz), 7.86 (dd, 1H, *J* = 8.1, 2.2 Hz), 8.25 (d, 1H, *J* = 2.2 Hz); ^13^C NMR (75 MHz, CDCl_3_): δ 28.1, 81.9, 83.0, 125.7, 131.1, 131.6, 132.2, 134.1, 134.4, 164.3, 165.1; HRMS (ESI^+^): Calcd for [M+Na]^+^, 379.0521; Found, 379.0552 (+3.1 mmu).

#### 2.2.3 Synthesis of 2,4-diCOOH DCTM

**Figure:**
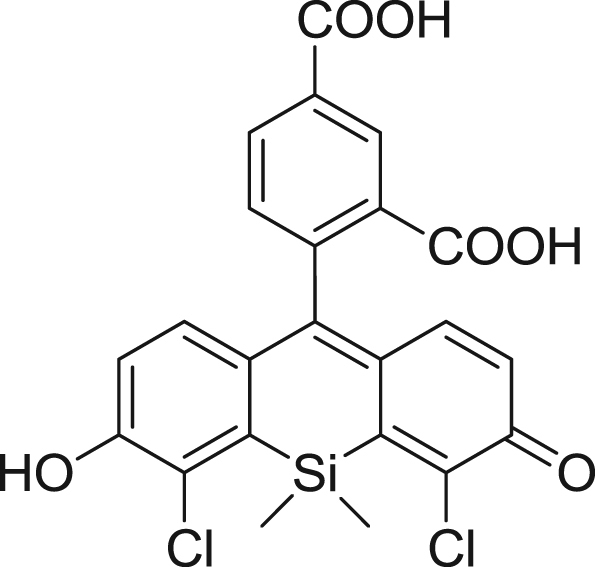

To a flame-dried flask flushed with argon, di-*tert*-butyl 4-bromoisophthalate (**2**) (357 mg, 1.00 mmol) and anhydrous THF (5 mL) were added. The solution was cooled to −78 °C, and 1 M *sec*-BuLi (0.70 mL, 0.70 mmol) was slowly added to it. The mixture was stirred at −78 °C for 10 seconds, then compound **1** (50.0 mg, 0.0705 mmol) dissolved in anhydrous THF (5 mL) was slowly added. The resulting mixture was warmed to room temperature, then stirred for 1 h, and 2 N HCl aq. (2.0 mL) was added to it. Stirring was continued for 20 min, then the mixture was extracted with CH_2_Cl_2_. The organic layer was washed with brine, dried over Na_2_SO_4_ and evaporated to dryness. The residue was dissolved in TFA (5.0 mL) and the solution was stirred for 1 h, then evaporated to dryness, and the resulting residue was purified by HPLC to give 2,4-diCOOH DCTM (18.1 mg, 53% yield). ^1^H NMR (300 MHz, CD_3_OD): δ 0.84 (s, 3H), 0.99 (s, 3H), 6.87 (d, 2H, *J*=8.8 Hz), 6.91 (d, 2H, *J* = 8.8 Hz), 7.05 (d, 1H, *J* = 8.1 Hz), 8.22 (dd, 1H, *J* = 8.1, 1.5 Hz), 8.49 (d, 1H, *J* = 1.5 Hz); ^13^C NMR (100 MHz, CD_3_OD): δ −0.1, 0.5, 91.4, 119.7, 124.0, 124.9, 127.8, 128.0, 128.3, 133.5, 135.6, 135.9, 137.5, 154.4, 162.3, 167.7, 171.9; HRMS (ESI^+^): Calcd for [M+H]^+^, 487.0171, Found, 487.0186 (+1.5 mmu).

#### 2.2.4 Synthesis of 5-amino-BAPTA-tetramethylester (**3**)

**Figure:**
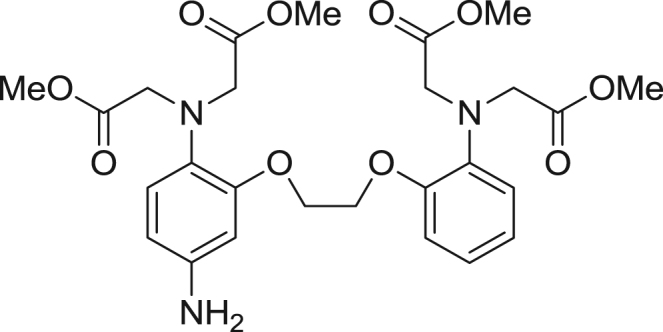

5-Amino-BAPTA-tetramethylester (**3**) was synthesized according to reference [3], [4].

#### 2.2.5 Synthesis of CaTM-3

**Figure:**
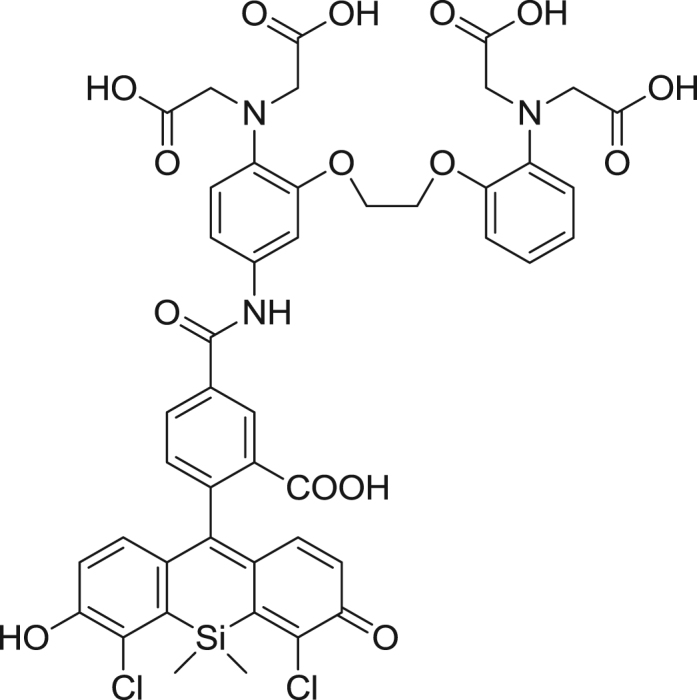

To a solution of 5-amino-BAPTA-tetramethylester (**3**) (27.4 mg, 0.0500 mmol) in DMF (3.0 mL), HATU (37.0 mg, 0.100 mmol), HOBt (15.3 mg, 0.100 mmol) and 2,4-diCOOH DCTM (9.8 mg, 0.020 mmol) were added. The mixture was stirred at room temperature overnight, then 2 N HCl aq. (2 mL) was added to it. The whole was extracted with CH_2_Cl_2_. The organic layer was washed with brine, dried over Na_2_SO_4_ and evaporated to dryness. Then, 2 N NaOH aq. (3.0 mL) and MeOH (3.0 mL) were added to the residue. The mixture was stirred at room temperature for 4 h, then neutralized with 2 N HCl aq., and purified by HPLC to give CaTM-3 (4.6 mg, 24% yield). ^1^H NMR (300 MHz, CD_3_OD): δ 0.84 (s, 3H), 1.00 (s, 3H), 3.74 (s, 4H), 3.77 (s, 4H), 4.38 (s, 4H), 6.84–6.94 (m, 9H), 7.09 (d, 1H, *J* = 8.1 Hz), 7.33 (d, 1H, *J* = 7.3 Hz), 7.39 (s, 1H), 8.14 (d, 1H, *J* = 8.1 Hz), 8.46 (s, 1H); HRMS (ESI^+^): Calcd for [M + H]^+^, 960.1606, Found, 960.1616 (+1.0 mmu). HPLC analysis: retention time 13.7 min (purity, 99.7% integrated intensity); eluent: A (H_2_O, 0.1 M TEAA (triethylammonium acetate)), B (80% acetonitrile/H_2_O, 0.1 M TEAA); gradient: A : B = 80 : 20 to 0 : 100 (15 min); flow rate: 1.0 mL/min; detection wavelength, 280 nm.

#### 2.2.6 Synthesis of 5-amino-BAPTA-tetraacetoxymethylester (**4**)

**Figure:**
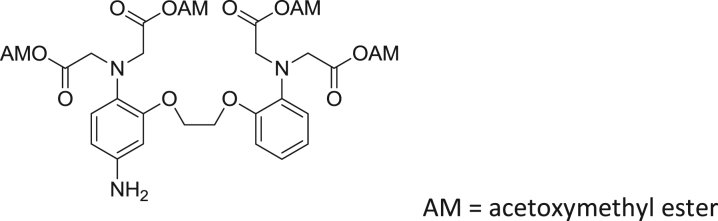

5-Amino-BAPTA-tetraacetoxymethylester (**4**) was synthesized according to Ref. [2].

#### 2.2.7 Synthesis of CaTM-3 AM

**Figure:**
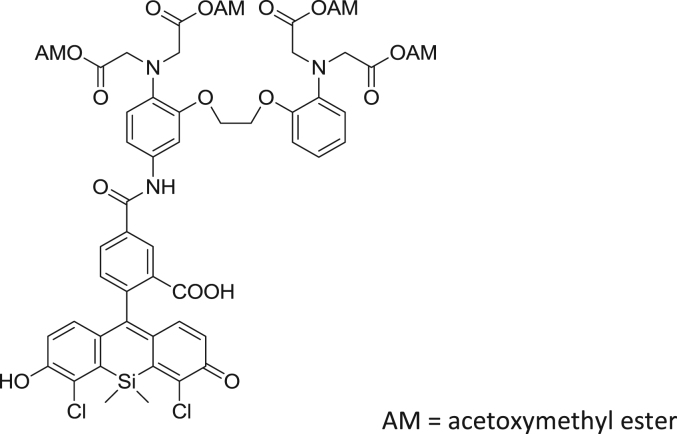

**Scheme 1:**
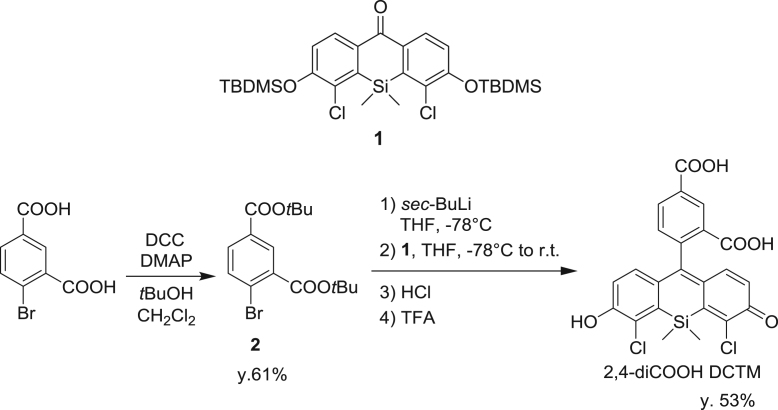
Synthetic scheme for 2,4-diCOOH DCTM.

**Scheme 2:**
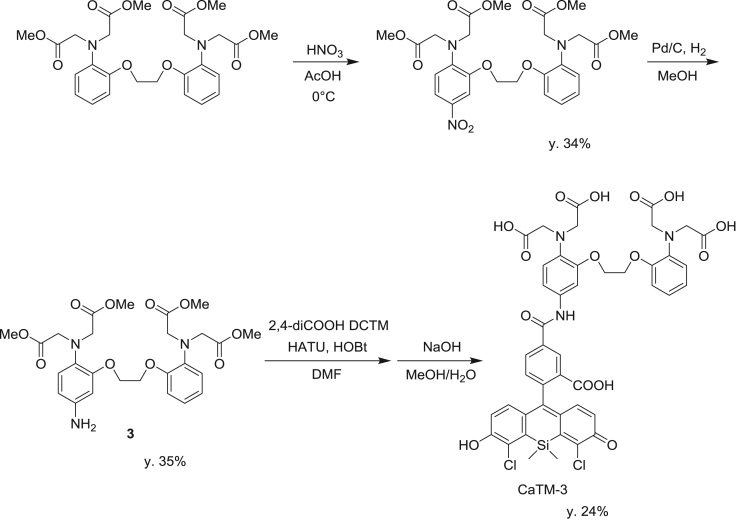
Synthetic scheme for CaTM-3.

**Scheme 3:**
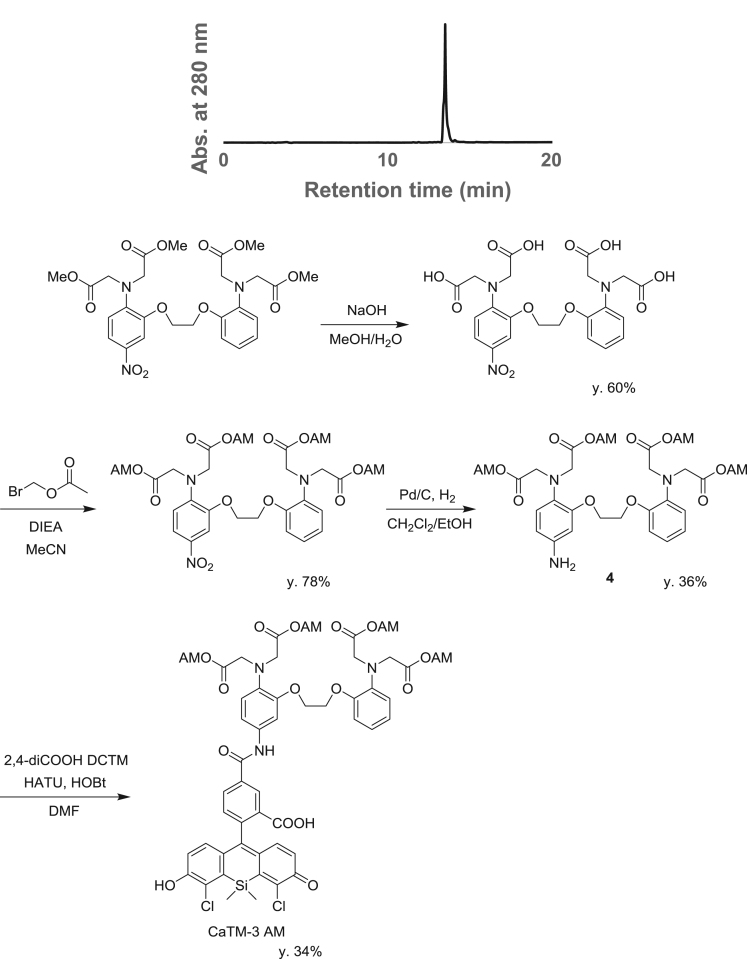
Synthetic Scheme for CaTM-3 AM (AM = acetoxymethyl ester).

To a solution of 5-amino-BAPTA-tetraacetoxymethylester (**4**) (31.2 mg, 0.0400 mmol) in DMF (2.0 mL), HATU (18.5 mg, 0.0500 mmol), HOBt (7.7 mg, 0.050 mmol) and 2,4-diCOOH DCTM (9.8 mg, 0.020 mmol) were added. The mixture was stirred at room temperature overnight, then neutralized with AcOH aq., and purified by HPLC to give CaTM-3 AM (8.6 mg, 34% yield). ^1^H NMR (300 MHz, CD_3_OD): δ 0.84 (s, 3H), 1.00 (s, 3H), 2.00 (s, 6H), 2.02 (s, 6H), 4.17 (s, 8H), 4.30 (s, 4H), 5.58 (s, 4H), 5.59 (s, 4H), 6.83–7.09 (m, 9H), 7.08 (d, 1H, *J* = 8.1 Hz), 7.17 (dd, 1H, *J* = 8.8, 2.2 Hz), 7.46 (d, 1H, *J*=2.2 Hz), 8.11 (dd, 1 H, *J* = 8.1, 1.5 Hz), 8.44 (s, 1 H); ^13^C NMR (100 MHz, CD_3_OD): δ −0.1, 0.5, 20.5, 54.6, 54.7, 68.6, 68.7, 80.6, 80.6, 91.4, 108.5, 114.9, 115.0, 119.7, 120.7, 120.8, 122.5, 123.8, 124.1, 124.9, 126.1, 127.9, 127.9, 134.8, 135.8, 135.9, 137.1, 137.8, 140.21, 152.1, 154.4, 161.2, 166.5, 171.1, 171.7, 177.8, 172.1; HRMS (ESI^+^): Calcd for [M + H]^+^, 1248.2451, Found, 1248.2420 (−3.1 mmu). (Scheme 1, Scheme 2, Scheme 3).
