# Supplementary material for: Synthesis of practical red fluorescent probe for cytoplasmic calcium ions with greatly improved cell-membrane permeability
Source: Data Brief. 2017 Apr 13;12:351–7. doi: 10.1016/j.dib.2017.04.011 (PMC5412011; doi:10.1016/j.dib.2017.04.011)
Supplement: Supplementary file 1 — Supplementary material [file mmc1.docx]

**Conflict of Interest**

The authors declare that there are no conflicts of interest.
